# Serum Levels of Kisspeptin Are Elevated in Patients with Pancreatic Cancer

**DOI:** 10.1155/2019/5603474

**Published:** 2019-10-20

**Authors:** Sven H. Loosen, Mark Luedde, Georg Lurje, Martina Spehlmann, Pia Paffenholz, Tom Florian Ulmer, Frank Tacke, Mihael Vucur, Christian Trautwein, Ulf P. Neumann, Tom Luedde, Christoph Roderburg

**Affiliations:** ^1^Department of Medicine III, University Hospital RWTH Aachen, Pauwelsstrasse 30, 52074 Aachen, Germany; ^2^Department of Internal Medicine III, University Hospital of Schleswig Holstein, Campus Kiel, Rosalind-Franklin-Str. 12, 24105 Kiel, Germany; ^3^Department of Visceral and Transplantation Surgery, University Hospital RWTH Aachen, Pauwelsstrasse 30, 52074 Aachen, Germany; ^4^Department of Urology, University Hospital Cologne, Cologne, Germany; ^5^Department of Hepatology and Gastroenterology, Charité University Medicine Berlin, Augustenburger Platz 1, 10117 Berlin, Germany; ^6^Department of Surgery, Maastricht University Medical Centre (MUMC), PO Box 5800, Maastricht, Netherlands; ^7^Division of Gastroenterology, Hepatology and Hepatobiliary Oncology, University Hospital RWTH Aachen, Pauwelsstrasse 30, 52074 Aachen, Germany

## Abstract

Pancreatic adenocarcinoma (PDAC) still represents a devastating disease associated with a very limited survival. Novel biomarkers allowing an early diagnosis as well as an optimal selection of suitable treatment options for individual patients are urgently needed to improve the dismal outcome of PDAC patients. Recently, alterations of Kisspeptin serum levels, a member of the adipokine family, were described in various types of cancers. However, the role of circulating Kisspeptin as a biomarker in PDAC patients is poorly defined. In this study, we measured Kisspeptin serum levels in a cohort of 128 prospectively enrolled PDAC patients undergoing surgical resection as well as 36 healthy controls. Kisspeptin concentrations were elevated in PDAC patients compared to control samples. Nevertheless, Kisspeptin serum levels were independent of tumor-related factors such as the tumor grading, TNM stage, or clinical features such as the ECOG performance status. Finally, in our analysis, neither preoperative nor postoperative Kisspeptin levels turned out as a significant predictor of overall survival after tumor resection. In conclusion, our data suggest that Kisspeptin concentrations are altered in PDAC patients but do not allow to predict patients' outcome after resection of PDAC.

## 1. Introduction

Despite recent advances in the surgical and systemic treatment of pancreatic adenocarcinoma (PDAC), this malignancy still represents a devastating disease [1, 2]. While the overall 5-year survival rate for all patients is still below 20%, radical tumor resection followed by adjuvant chemotherapy can result in a long term for selected patients with early disease stages [3, 4]. However, only 8 to 16% of all PDAC patients represent potential candidates for a curatively intended tumor resection [3, 4], as most patients initially present with an advanced, nonresectable tumor stage at the time of diagnosis [3, 5]. Moreover, many resected PDAC patients are facing an early tumor recurrence, thus leaving these patients without any benefit from the extensive tumor resection [6]. Together, improving the rate of patients who are diagnosed with a resectable disease stage and identifying patients who will particularly benefit from extensive tumor resection might help to improve the patients' prognosis. Besides standard diagnostic approaches such as imaging techniques, easily accessible biomarkers might yield important information on individual patient's disease characteristics and will thus be of increasing importance for both of these issues [7, 8].

Kisspeptin, first described in 1996 by Lee and Welch in melanoma cells, belongs to the adipokine family [9]. High expression levels of Kisspeptin have been detected in the placenta [10, 11] as well as other tissues including the pancreas, liver, and skeletal muscle [12, 13], arguing for a decisive role of Kisspeptin in metabolism and homeostasis. Moreover, several authors suggested an antimetastatic and antitumoral role of Kisspeptin in different malignancies such as bladder, ovarian, colorectal, prostate, and thyroid cancer [14]. As such, Kisspeptin was shown to inhibit colorectal cancer cell invasiveness by binding to the KISS1R receptor and thus activating PKR and PP2A [15]. With respect to pancreatic cancer, it was demonstrated that pancreatic tumors that did not (or only at very low levels) express Kisspeptin were significantly larger in size when compared to Kisspeptin-positive tumors [16]. Moreover, PDAC patients with a high intratumoral Kisspeptin expression displayed lower disease recurrence rates after tumor resection compared to Kisspeptin-negative tumor patients, and a strong expression of Kisspeptin correlated with a longer overall survival [16]. However, only very limited data regarding a potential role of Kisspeptin serum levels as a surrogate for tumor characteristics or the patients' outcome for PDAC patients are available to date [17]. Thus, to further evaluate the hypothesis of a potential diagnostic and/or prognostic role of circulating Kisspeptin in the context of PDAC, we determined the serum level of Kisspeptin in a large cohort of 128 patients with PDAC who underwent surgical tumor resection.

## 2. Materials and Methods

### 2.1. Design of Study and Patient Cohort

In this study, we evaluated circulating levels of Kisspeptin as a novel diagnostic and/or prognostic biomarker in a cohort of 128 PDAC patients who underwent surgical tumor resection at the University Hospital RWTH Aachen between 2011 and 2016 (see Table 1). Patients with PDAC manifestation in the pancreatic head received either Whipple procedure or pylorus-sparing pancreaticoduodenectomy (PPPD), while patients with tumor manifestation in the tail or body of the pancreas received distal pancreatectomy. The presence of PDAC was confirmed histopathologically after tumor resection. Patients' blood samples were collected within 24 hours before surgery as well as 6 to 7 days after PDAC resection. After collection, blood samples were centrifuged for 10 min at 2000 g, and serum aliquots of 1 ml were frozen immediately at -80°C in order to avoid repetitive freeze-thaw cycles until use. 36 healthy, cancer-free blood donors served as control samples. The study protocol was approved by the local ethics committee and conducted in accordance with the ethical standards laid down in the Declaration of Helsinki (EK 206/09, ethics committee of the University Hospital Aachen, RWTH University, Aachen, Germany). Written informed consent was obtained from the patients.

### 2.2. Measurement of Circulating Kisspeptin Levels

Circulating levels of Kisspeptin were measured with an enzyme-linked immunosorbent assay (ELISA) according to the manufacturers' instructions (Human Kisspeptin 1 (KISS1) ELISA Kit, Abbexa Ltd., Cambridge, United Kingdom, No.: abx152134). The Kisspeptin 1 Kit is a sandwich ELISA kit for use with serum and plasma. There are no significant cross reactivity or interference between Kisspeptin 1 (KISS1) and analogues. Serum samples were measured without previous dilution.

### 2.3. Statistical Analysis

Statistical analyses were performed as previously described [18]. Kisspeptin concentrations are displayed as median and range. The Shapiro-Wilk test was applied to test for normal distribution of value, and the Mann-Whitney *U* test was used when normal distribution could not be proved. The Kruskal-Wallis test was used for comparisons with more than two groups. Box plot graphics display the median, quartiles, and ranges. In ROC curve analysis, sensitivity was plotted against 1 − specificity. The Kaplan-Meier curve analysis was used for survival analysis in combination with the log-rank test to test for differences. The optimal Kisspeptin cut-off value with respect to the patients' overall survival was calculated using publicly available software [19]. All statistical analyses were performed with SPSS 23 (SPSS, Chicago, IL, USA) [20].

## 3. Results

### 3.1. Kisspeptin Concentrations Are Elevated in Patients with PDAC

**Figure 1:**
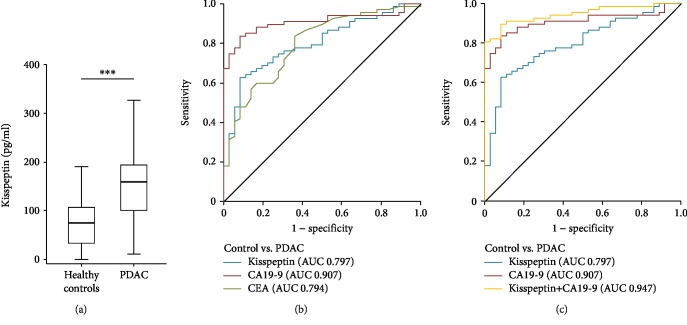
Serum Kisspeptin levels are significantly elevated in patients with PDAC. (a) Serum levels of Kisspeptin were determined by ELISA in PDAC patients and compared to those in healthy controls. (b) CA19-9 shows the highest AUC value regarding the diagnosis of PDAC. (c) ROC analysis comparing the diagnostic relevance of Kisspeptin and CA-19-9 and the combination of Kisspeptin and CA19-9 for PDAC.

Results from previous publications suggest that PDAC patients with high tumoral Kisspeptin expression display a more favorable outcome compared to patients with low Kisspeptin tissue levels [16]. Therefore, we measured preoperative Kisspeptin serum concentrations in 128 patients who underwent surgical PDAC tumor resection (Tables 1 and 2) and compared them to healthy controls. Interestingly, this analysis revealed significantly elevated levels of circulating Kisspeptin in PDAC patients compared to control samples (Figure 1(a)). In ROC curve analysis, Kisspeptin showed an AUC value of 0.797 for the differentiation between PDAC patients and healthy control samples (Figure 1(b)). At an ideal cut-off value of 140.9 pg/ml that was established using the Youden index method, Kisspeptin showed a sensitivity of 62.7% with a specificity of 91.7% for the diagnosis of PDAC. Next, we compared the diagnostic potential of circulating Kisspeptin with clinically established PDAC tumor markers such as CA19-9 and CEA. Interestingly, in this analysis, the diagnostic power of Kisspeptin was numerically lower than CA19-9 but slightly higher compared to CEA (AUC_CA19-9_: 0.907, AUC_CEA_: 0.794, Figure 1(b)). Of note, when the combination of Kisspeptin and CA19-9 was tested in this context, the diagnostic potential was even superior compared to each marker alone (AUC_Kisspeptin/CA19-9_: 0.947, Figure 1(c)). In this setting, the combination of CA19-9 and Kisspeptin revealed a superior diagnostic sensitivity/specificity of 89.6/91.7%.

### 3.2. Circulating Kisspeptin Does Not Reflect Disease Characteristics

Based on these promising results, we subsequently attempted to analyze whether serum concentrations of Kisspeptin might reflect disease-specific clinicopathological parameters such as the TNM stage, the tumor resection status (R_0_ vs. R_1_), the histological tumor grading (G_2_ vs. G_3_), or the patients clinical performance status (ECOG 0 vs. ECOG ≥ 1). We therefore performed subgroup analyses and specifically analyzed Kisspeptin levels in patients with early or more advanced T-stages (Suppl. Fig. [Supplementary-material supplementary-material-1]), nodal-negative or nodal-positive tumor (Suppl. Fig. [Supplementary-material supplementary-material-1]), and nonmetastasized or metastasized patients who were still eligible for tumor resection (Suppl. Fig. [Supplementary-material supplementary-material-1]). However, preoperative Kisspeptin levels were unaltered in all analyzed subgroups. Next, we analyzed whether preoperative Kisspeptin concentrations might reflect the postoperative resection status (R_0_ vs. R_1_, Suppl. Fig. [Supplementary-material supplementary-material-1]) or the tumor grading (G_2_ vs. G_3_, Suppl. [Supplementary-material supplementary-material-1]). However, also in these analyses, no significant differences in Kisspeptin concentrations became apparent. Finally, preoperative serum Kisspeptin levels did not reflect the patients' ECOG PS (Suppl. Fig. [Supplementary-material supplementary-material-1]).

To further unravel potential mechanisms regulating Kisspeptin serum concentrations in the context of PDAC, we subsequently performed extensive correlation analyses between Kisspeptin and various routine markers of organ dysfunction (supplementary [Supplementary-material supplementary-material-1]). These analyses revealed that preoperative Kisspeptin concentrations do not correlate with markers of systemic inflammation (leukocyte count and CRP), liver function (AST, ALT, ALP, GGT, and bilirubin), PDAC tumor markers (CEA, CA19-9), or other standard laboratory parameters (supplementary [Supplementary-material supplementary-material-1]). However, Kisspeptin serum levels correlated with the patients' renal function (creatinine: *R*: 0.289, *p* = 0.001, supplementary [Supplementary-material supplementary-material-1]).

### 3.3. Preoperative Serum Kisspeptin Levels Do Not Predict Survival after Tumor Resection

**Figure 2:**
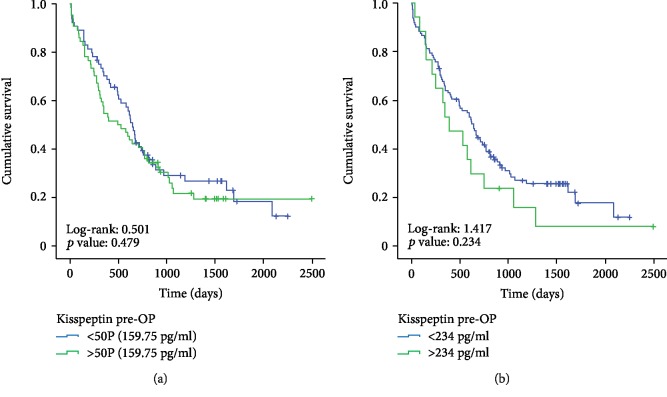
Preoperative Kisspeptin concentrations do not reflect the patients' prognosis following PDAC resection. (a) The 50^th^ percentile (159.75 pg/ml) of preoperative Kisspeptin serum levels is no suitable discriminator to identify patients who display long-term survival. (b) The optimal preoperative Kisspeptin cut-off value (234.0 pg/ml) is no discriminator between patients who display long-term survival and those who did not.

Recently, Nagai et al. demonstrated that intratumoral Kisspeptin expression might be predictive for the patients' survival after complete pancreatectomy [16]. Based on these data, we next attempted to identify whether preoperative Kisspeptin serum levels might also be indicative for the patients' survival after tumor resection. We therefore divided our series of patients into those with serum Kisspeptin levels above the 50^th^ percentile (159.75 pg/ml) and those patients with Kisspeptin serum concentration below 159.75 pg/ml. Notably, we failed to detect differences in outcome for either of the two groups (Figure 2(a)). Next, we hypothesized that a calculated “optimal cut-off value” for Kisspeptin concentrations might be more suitable as a discriminator and determined the ideal prognostic Kisspeptin cut-off value (234.0 pg/ml) as recently described for subsequent Kaplan-Meier curve analysis [19, 21]. Nevertheless, patients with Kisspeptin serum levels above 234.0 pg/ml showed a similar long-term survival compared to patients with Kisspeptin serum levels below the ideal cut-off value (Figure 2(b)). In line, Cox regression analysis revealed that initial Kisspeptin concentration above 234 pg/ml was unsuitable to predict the patient's overall survival after tumor resection (hazard ratio: 1.396 (0.804-2.427), *p* = 0.236).

### 3.4. Postoperative Kisspeptin Serum Concentrations and Outcome of Patients

**Figure 3:**
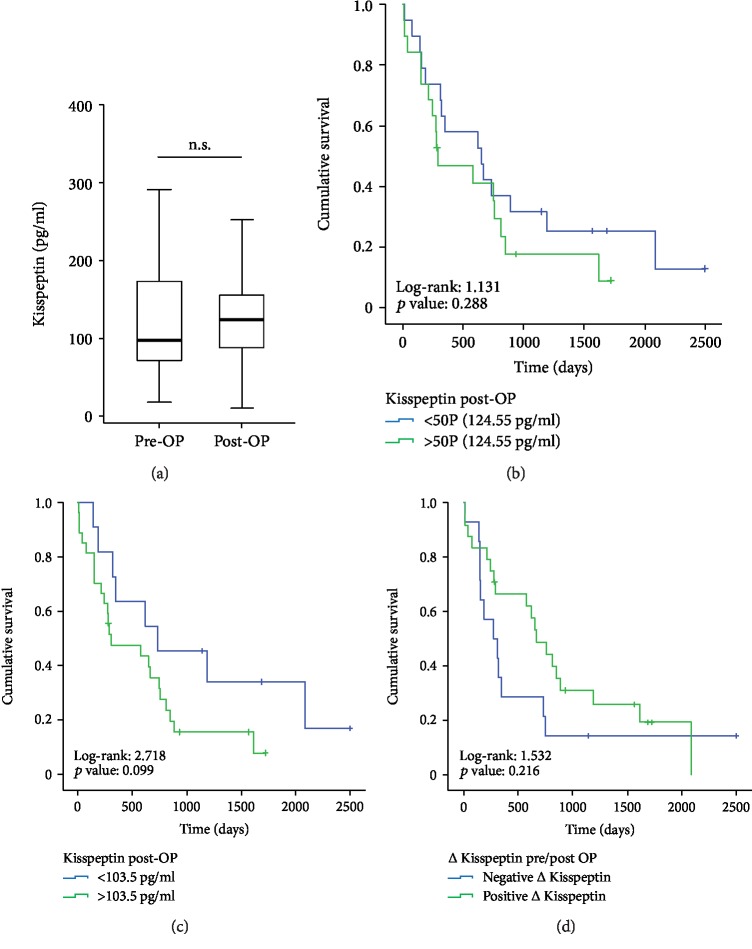
Postoperative Kisspeptin concentrations and patients' outcome. (a) Postoperative concentrations of Kisspeptin are similar to those measured before surgery. (b) The 50^th^ percentile of postoperative Kisspeptin concentrations (124.55 pg/ml) is no suitable discriminator to identify patients who display long-term survival and those who did not. (c) The ideal cut-off value for postoperative Kisspeptin concentrations (103.5 pg/ml) is no significant discriminator to identify patients who display long-term survival and those who did not (*p* = 0.099). (d) The individual course of Kisspeptin levels before and after tumor resection did not reflect patients' outcome after surgery.

Finally, for a subgroup of patients, we analyzed Kisspeptin concentrations at a later time point (6-7 days after tumor resection, *n* = 38). Of note, postoperative levels of circulating Kisspeptin were higher compared to healthy controls but unaltered when compared to the patients' respective preoperative levels in our cohort of PDAC patients (Figure 3(a)). Next, we analyzed if postoperative Kisspeptin concentrations might be indicative for the patients' outcome after tumor resection. However, neither the 50^th^ percentile (124.55 pg/ml) nor the calculated ideal cut-off value for postoperative Kisspeptin levels (103.5 pg/ml) was suitable to discriminate between patients who showed long-term survival and those who did not (Figures 3(b) and 3(c)). In line, Cox regression analysis revealed no prognostic relevance for elevated postoperative Kisspeptin concentrations above the ideal cut-off value (hazard ratio: 2.022 (0.860-4.753), *p* = 0.107). Finally, we hypothesized that the individual course of Kisspeptin serum level pre- and postsurgery might be a prognostic factor and compared the overall survival of patients with an increasing Kisspeptin level after tumor resection (*n* = 24) to patients with decreasing Kisspeptin levels (*n* = 14). However, the individual course of Kisspeptin serum levels before and after tumor resection did not turn out as a predictor for the patients' survival (Figure 3(d)).

## 4. Discussion

Alterations in serum concentrations of adipokines, including Kisspeptin, were recently described for various types of cancer [14]. In the present study, we demonstrate in a large cohort of PDAC patients who underwent tumor resection in a curative attempt that circulating levels of Kisspeptin are elevated in PDAC patients both at the time point of diagnosis and after tumor resection. This finding is in good agreement with results from an exploratory study that evaluated plasma Kisspeptin/metastin levels in a small cohort of patients with pancreatic cancer [17]. Although existing data on a relevant association of Kisspeptin serum levels and PDAC are very limited, several functional studies have suggested a role of Kisspeptin in the pathophysiology of PDAC. By using SCID mice implanted orthotopically with pancreatic cancer cells, McNally and colleagues demonstrated that the overexpression of Kisspeptin was associated with smaller tumors and prevented the development of both lung and liver metastases [22]. In line, Kisspeptin suppressed the migration of PANC-1 cells by activating ERK1, a kinase with a known role in pancreatic cancer [23]. Consequently, a strong tissue expression of Kisspeptin was found to be associated with a longer survival in PDAC patients, and intratumoral Kisspeptin expression turned out as a prognostic factor for overall survival in these patients [16]. While serum Kisspeptin levels tended to decrease in patients with advanced PDAC (T4, M1 patients; see Supplementary [Supplementary-material supplementary-material-1]) in our study, concentrations of circulating Kisspeptin did not significantly reflect clinicopathological disease parameters such as the tumor size, invasion of lymph nodes, or the presence of liver metastases. Moreover, in contrast to intratumoral Kisspeptin expression, serum levels of Kisspeptin did not correlate with the patients' outcome after surgical tumor resection as patients who died early during follow-up displayed similar preoperative Kisspeptin concentrations compared to long-term survivors (see Figures 2(a) and 2(b)). In this line of thinking, it would also be of interest to directly compare tissue expression and serum levels of Kisspeptin to gain further insight into a potential prognostic relevance of Kisspeptin in the context of PDAC. However, these analyses were not within the scope of this study.

Based on the strongly elevated circulating concentrations of Kisspeptin in PDAC patients (see Figure 1), we hypothesized that Kisspeptin levels might decline after tumor resection. However, in our cohort of PDAC patients, postoperative Kisspeptin levels were almost identical to those before tumor resection. This finding suggests that the tumor itself does not represent the most prominent source of circulating Kisspeptin in PDAC patients. Just recently, alterations of Kisspeptin levels were found in PBMCs from rats after Complete Freund's Adjuvant- (CFA-) induced orofacial inflammation [24]. Interestingly, in our study, we found a trend towards a positive correlation of Kisspeptin levels and serum concentrations of C-reactive protein (CRP) as a surrogate for systemic inflammation (see Supplementary [Supplementary-material supplementary-material-1]).

Radical tumor resection is the only potentially curative treatment modality in the context of PDAC. However, only about 12% of all patients might be considered for tumor resection [5], since most patients present with nonresectable disease stage at the time of diagnosis [3]. In this context, easily accessible markers, allowing the diagnosis of PDAC already in early or even very early clinical stages might help to improve the patients' prognosis by increasing the percentage of patients who will undergo complete tumor resection. Here, we show that Kisspeptin serum concentrations are elevated in patients with PDAC. Despite the fact that the diagnostic potential of serum Kisspeptin alone was inferior to CA19-9, the combination of both markers revealed a superior discriminatory power for the diagnosis of PDAC, arguing that Kisspeptin might be implemented into a novel diagnostic PDAC tumor marker panel. In this line of thinking, it would also be interesting to evaluate Kisspeptin serum concentrations in clinical conditions that favor the development of PDAC such as chronic pancreatitis or even PDAC precursor lesions such as IPMN and PanIN lesions to further narrow down the specificity of elevated Kisspeptin levels in PDAC patients.

In summary, our data suggest that serum Kisspeptin measurements might be useful as an additional tool in the complex diagnosis work-up of PDAC. However, the results are limited by the fact that we only included patients from a single center cohort into our analyses. Thus, further longitudinal studies using independent validation cohorts of PDAC patients need to confirm our results before a use in clinical routine can be considered. Nevertheless, our data on Kisspeptin concentrations in PDAC are not only interesting from a clinical point of view but also provide evidence for a functional role of Kisspeptin in pancreatic cancer and might stimulate further research on the role of this adipokine in the context of PDAC.

## Figures and Tables

**Table 1:** Patient characteristics of study cohort.

PDAC patients	128
Sex (%)	
Male-female	62.8-37.2
Age (years, median and range)	68.0 (42 - 84)
BMI (kg/m^2^, median and range)	24.64 (16.24 - 43.21)
PDAC characteristics (%)	
T_1_-T_2_-T_3_-T_4_	3.7-3.7-86.9-5.6
N_0_-N_1_	29.9-70.1
M_0_-M_1_	82.9-17.1
G_2_-G_3_	52.0-48.0
R_0_-R_1_	68.4-31.6
Clinical performance status (%)	
ECOG 0-1-2-3	51.8-34.2-9.6-4.4
Operation technique	
Pylorus-sparing pancreaticoduodenectomy (PPPD)/Whipple procedure	89.8
Distal pancreatectomy	10.2
Deceased during follow-up (%)	
Yes-no	74.4-25.6

**Table 2:** Levels of various laboratory parameters.

	PDAC patients Median (range)	Healthy controls Median (range)
Kisspeptin pre-OP (pg/ml)	159.75 (11.3-432.4)	79.81 (0.91-232.3)
Kisspeptin post-OP (pg/ml)	124.55 (11.29-272.9)	
CEA (*μ*g/l)	2.9 (0.22-76.30)	1.3 (0.3-6.3)
CA 19-9 (U/ml)	115.8 (0.6-5637.0)	5.7 (0.0-44.1)
Sodium (mmol/l)	139.0 (121.0-149.0)	
Potassium (mmol/l)	4.3 (2.8-5.8)	
Leucocyte count (cells/nl)	7.5 (2.7-22.1)	
Platelets (cells/nl)	267.0 (117.0-799.0)	
CRP (mg/l)	8.25 (0.0-237.0)	
AST (U/l)	32.0 (13.0-405.0)	30.0 (20.0-78.0)
ALT (U/l)	41.0 (7.0-651.0)	24.0 (5.0-82.0)
GGT (U/l)	131.0 (10.0-2138.0)	18.8 (8.0-98.0)
ALP (U/l)	133.0 (42.0-1574.0)	68.0 (40.0-100.0)
LDH	179.5 (113.0-317.0)	160.0 (60.0-204.0)
Bilirubin (mg/dl)	0.69 (0.2-26.2)	0.44 (0.10-1.46)
Creatinine (mg/dl)	0.855 (0.4-3.3)	

CEA: carcinoembryonic antigen, CA 19-9: carbohydrate-antigen 19-9, CRP: C-reactive protein, AST: aspartate transaminase, ALT: alanine transaminase, GGT: *γ*-glutamyl transpeptidase, ALP: alkaline phosphatase, LDH: lactate dehydrogenase.

## Data Availability

Data included into this analysis represent highly sensitive personal medical data. It is directly against German (and European) law to publish such data in a way that would allow identifying individual patients (e.g., by providing different clinical values of one distinct patient). Data are available upon request from the Department of Medicine III of the University Hospital RWTH Aachen for researchers who meet the criteria for access to confidential data (med3@ukaachen.de).

## Supplementary Materials

Supplementary Materials Supplementary Figure 1: Kisspeptin serum levels before tumor resection do not reflect tumor characteristics. Circulating levels of Kisspeptin are unaltered between PDAC patients with different T-stages (A), N_0_ vs. N_1_ tumor stage (B) and M_0_ vs. M_1_ disease stage (C). Kisspeptin levels are comparable between patients with complete tumor resection (R_0_) and patients with microscopically positive tumor margins (R_1_, D) as well as PDAC patients with moderately (G_2_) or poorly (G_3_) differentiated tumors (E). Kisspeptin levels do not reflect the patients' ECOG performance status (F).
