# Supplementary material for: Serum Levels of Kisspeptin Are Elevated in Patients with Pancreatic Cancer
Source: Dis Markers. 2019 Oct 20;2019:5603474. doi: 10.1155/2019/5603474 (PMC6854939; doi:10.1155/2019/5603474)
Supplement: Supplementary Materials — Supplementary Figure 1: Kisspeptin serum levels before tumor resection do not reflect tumor characteristics. Circulating levels of Kisspeptin are unaltered between PDAC patients with different T-stages (A), N0 vs. N1 tumor stage (B) and M0 vs. M1 disease stage (C). Kisspeptin levels are comparable between patients with complete tumor resection (R0) and patients with microscopically positive tumor margins (R1, D) as well as PDAC patients with moderately (G2) or poorly (G3) differentiated tumors (E). Kisspeptin levels do not reflect the patients' ECOG performance status (F). [file 5603474.f1.docx]

**Supplementary Figures**


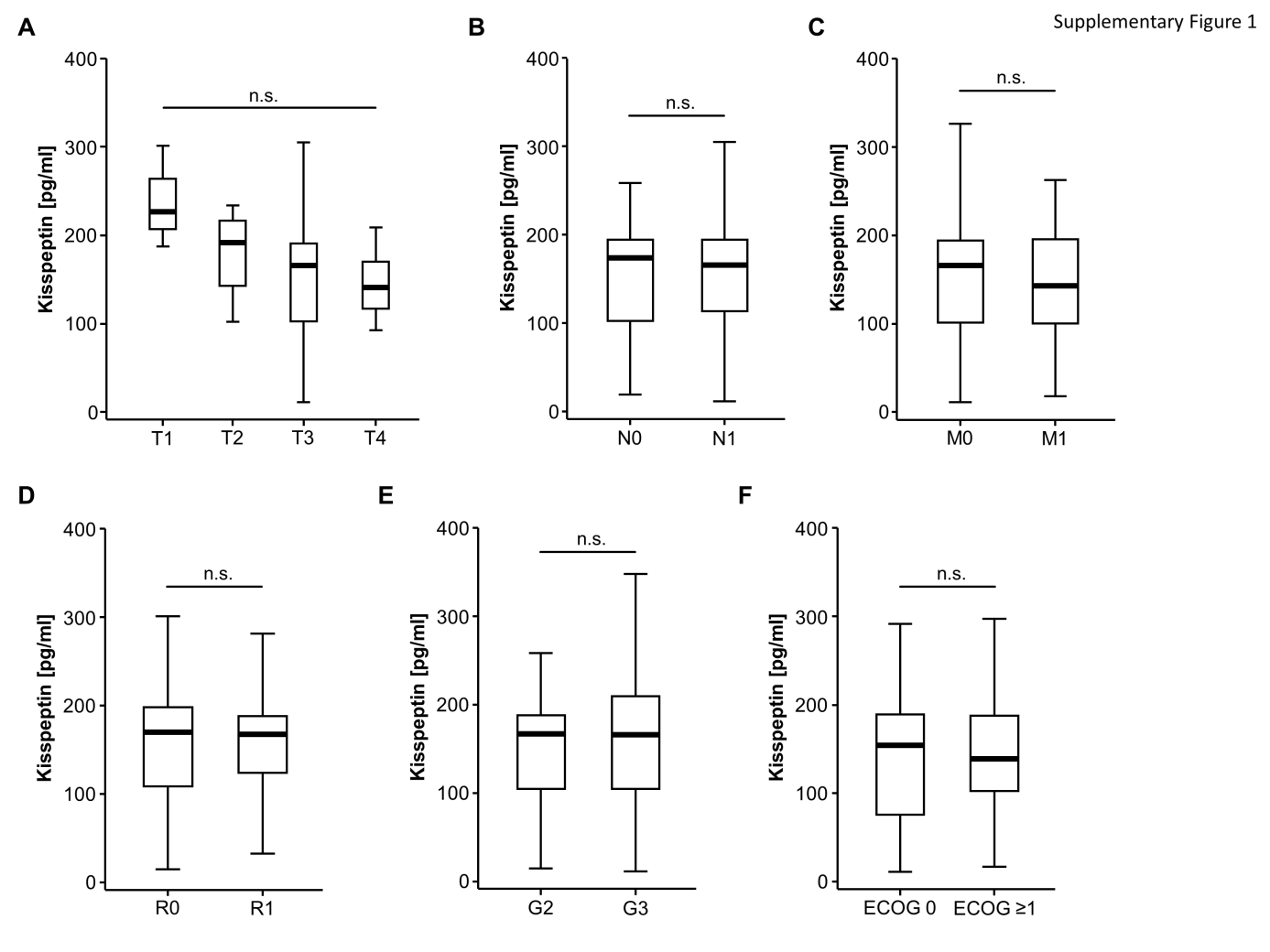


***Supplementary Figure 1. Kisspeptin serum levels before tumor resection do not reflect tumor characteristics.*** *Circulating levels of Kisspeptin are unaltered between PDAC patients with different T-stages (A), N0 vs. N1 tumor stage (B) and M0 vs. M1 disease stage (C). Kisspeptin levels are comparable between patients with complete tumor resection (R0) and patients with microscopically positive tumor margins (R1, D) as well as PDAC patients with moderately (G2) or poorly (G3) differentiated tumors (E). Kisspeptin levels do not reflect the patients´ ECOG performance status (F).*

**Supplementary Table 1.** Correlation analysis between Kisspeptin and various laboratory parameters

| **Parameter** | **Kisspeptin** | |
| --- | --- | --- |
|  | **R** | **p-value** |
| Sodium | -0.049 | 0.586 |
| Potassium | 0.015 | 0.868 |
| Haemoglobin | -0.156 | 0.078 |
| Leukocytes | 0.076 | 0.391 |
| Thrombocytes | -0.102 | 0.252 |
| AST | -0.030 | 0.739 |
| ALT | 0.032 | 0.883 |
| LDH | 0.007 | 0.975 |
| Bilirubin | 0.056 | 0.530 |
| GGT | 0.080 | 0.396 |
| ALP | 0.047 | 0.618 |
| CRP | 0.168 | 0.076 |
| Creatinine | 0.289* | 0.001 |
| CEA | 0.201 | 0.100 |
| CA19-9 | 0.170 | 0.151 |

AST: aspartate transaminase, ALT: alanine transaminase, LDH: lactate dehydrogenase, GGT: γ-Glutamyl transpeptidase, ALP: alkaline phosphatase, CRP: C-reactive protein, CEA: carcinoembryonic antigen, CA 19-9: carbohydrate-Antigen 19-9, * p<0.05
